# Supplementary material for: Effects of Exercise on Inflammatory Markers in Individuals with Chronic Kidney Disease: A Systematic Review and Meta-Analysis
Source: Metabolites. 2023 Jun 27;13(7):795. doi: 10.3390/metabo13070795 (PMC10385645; doi:10.3390/metabo13070795)
Supplement: Supplementary file 1 [file metabolites-13-00795-s001.zip › metabolites-2440244-supplementary.pdf]

SUPPLEMENTARY DATA

Table S1. Search strategy

Data base	Terms
MEDLINE	(((Kidney Failure, Chronic[Title/Abstract]) OR (Chronic Kidney Failure[Title/Abstract])) OR (End-Stage Renal Disease[Title/Abstract])) OR (End-Stage Renal Failure[Title/Abstract])) OR (Renal Failure, End-Stage[Title/Abstract])) OR (Renal Failure, Chronic[Title/Abstract])) OR (ESRD[Title/Abstract])) OR (Renal Insufficiency, Chronic[Title/Abstract])) OR (Renal Insufficiency[Title/Abstract])) OR (Chronic Renal Insufficiency[Title/Abstract])) OR (Kidney Insufficiency[Title/Abstract])) OR (Kidney Failure[Title/Abstract])) OR (Renal Failure[Title/Abstract])) OR (Chronic Kidney Disease[Title/Abstract])) OR (Chronic Renal Disease[Title/Abstract])) OR (Acute kidney injury[Title/Abstract])) OR (Acute Renal Injury[Title/Abstract])) OR (Acute Kidney Insufficiency[Title/Abstract])) OR (Acute Renal Failure[Title/Abstract])) OR (Acute Kidney Failure[Title/Abstract])) OR (Cardio-Renal Syndrome[Title/Abstract])) OR (Renal

	HD[Title/Abstract])) OR (HemoHD[Title/Abstract])) OR (Extracorporeal HD[Title/Abstract])) OR (Hemodiafiltration[Title/Abstract])) OR (HemoHD, Home[Title/Abstract])) OR (Renal HD, Home[Title/Abstract])) OR (Home Renal HD[Title/Abstract])) OR (Home HemoHD[Title/Abstract])) OR (Peritoneal HD[Title/Abstract])) OR (HD, Peritoneal[Title/Abstract])) OR (Peritoneal HD, Continuous Ambulatory[Title/Abstract])) OR (Continuous Ambulatory Peritoneal HD[Title/Abstract])) OR (Renal Replacement Therapy[Title/Abstract])) OR (Kidney Replacement Therapy[Title/Abstract])) OR (Continuous Renal Replacement Therapy[Title/Abstract])) OR (Intermittent Renal Replacement Therapy[Title/Abstract])) OR (Hemofiltration[Title/Abstract])) OR (Hemoperfusion[Title/Abstract])) OR (Hybrid Renal Replacement Therapy[Title/Abstract])) AND (randomizedcontrolledtrial[Filter])) AND (((Exercise[Title/Abstract]) OR (Exercises[Title/Abstract])) OR (Physical Activity[Title/Abstract])) OR (Physical Activities[Title/Abstract])) OR (Physical Exercise[Title/Abstract])) OR (Acute Exercise[Title/Abstract])) OR (Isometric Exercise[Title/Abstract])) OR (Aerobic

	Exercise[Title/Abstract])) OR (Exercise Training[Title/Abstract])) OR (Cool-Down Exercise[Title/Abstract])) OR (Cool Down Exercise[Title/Abstract])) OR (Warming-Down Exercise[Title/Abstract])) OR (Warming Down Exercise[Title/Abstract])) OR (Warm-Down Exercise[Title/Abstract])) OR (Warm Down Exercise[Title/Abstract])) OR (Cooldown Exercise[Title/Abstract])) OR (Cooling-Down Exercise[Title/Abstract])) OR (Cooling Down Exercise[Title/Abstract])) OR (Gymnastics[Title/Abstract])) OR (Calisthenics[Title/Abstract])) OR (Muscle Stretching Exercises[Title/Abstract])) OR (Static Stretching[Title/Abstract])) OR (Active Stretching[Title/Abstract])) OR (Static-Active Stretching[Title/Abstract])) OR (Static Active Stretching[Title/Abstract])) OR (Isometric Stretching[Title/Abstract])) OR (Ballistic Stretching[Title/Abstract])) OR (Dynamic Stretching[Title/Abstract])) OR (Proprioceptive Neuromuscular Facilitation (PNF) Stretching[Title/Abstract])) OR (Proprioceptive Neuromuscular Facilitation[Title/Abstract])) OR (Passive Stretching[Title/Abstract])) OR (Relaxed Stretching[Title/Abstract])) OR (Static-Passive Stretching[Title/Abstract])) OR (Static Passive Stretching[Title/Abstract])) OR (Physical Conditioning, Human[Title/Abstract])) OR (Human Physical

	Conditioning[Title/Abstract])) OR (Human Physical Training[Title/Abstract])) OR (Circuit-Based Exercise[Title/Abstract])) OR (Circuit Based Exercise[Title/Abstract])) OR (Circuit Training[Title/Abstract])) OR (Endurance Training[Title/Abstract])) OR (High-Intensity Interval Training[Title/Abstract])) OR (High Intensity Interval Training[Title/Abstract])) OR (High-Intensity Intermittent Exercise[Title/Abstract])) OR (Sprint Interval Training[Title/Abstract])) OR (Plyometric Exercise[Title/Abstract])) OR (Plyometric Drill[Title/Abstract])) OR (Plyometric Training[Title/Abstract])) OR (Stretch-Shortening Exercise[Title/Abstract])) OR (Stretch Shortening Exercise[Title/Abstract])) OR (Stretch-Shortening Cycle Exercise[Title/Abstract])) OR (Stretch Shortening Cycle Exercise[Title/Abstract])) OR (Stretch-Shortening Drill[Title/Abstract])) OR (Stretch Shortening Drill[Title/Abstract])) OR (Resistance Training[Title/Abstract])) OR (Strength Training[Title/Abstract])) OR (Weight-Lifting Strengthening Program[Title/Abstract])) OR (Weight Lifting Strengthening Program[Title/Abstract])) OR (Weight-Lifting Exercise Program[Title/Abstract])) OR (Weight Lifting Exercise Program[Title/Abstract])) OR (Weight-Bearing Strengthening Program[Title/Abstract])) OR (Weight Bearing Strengthening

	Program[Title/Abstract])) OR (Weight-Bearing Exercise Program[Title/Abstract])) OR (Weight Bearing Exercise Program[Title/Abstract])) OR (Exercise Therapy[Title/Abstract])) OR (Remedial Exercise[Title/Abstract])) OR (Remedial Exercises[Title/Abstract])) OR (Exercise Therapies[Title/Abstract])) OR (Rehabilitation Exercise[Title/Abstract])) OR (Rehabilitation Exercises[Title/Abstract])) OR (Running[Title/Abstract]))) OR (Swimming[Title/Abstract])) OR (Walking[Title/Abstract])) OR (Ambulation[Title/Abstract])) OR (Stair Climbing[Title/Abstract])) OR (Stair Navigation[Title/Abstract])) OR (Warm-Up Exercise[Title/Abstract])) OR (Warm Up Exercise[Title/Abstract])) OR (Warmup Exercise[Title/Abstract])) OR (Warming-Up Exercise[Title/Abstract])) OR (Warming Up Exercise[Title/Abstract])) OR (Motor Activity[Title/Abstract])) OR (Movement[Title/Abstract]) AND (randomizedcontrolledtrial[Filter]))) AND (((Inflammation[Title/Abstract]) OR (Innate Inflammatory Response[Title/Abstract])) OR (Interleukins[Title/Abstract])) OR (Interleukin-1[Title/Abstract])) OR (Interleukin-1alpha[Title/Abstract])) OR (Interleukin-

	1beta[Title/Abstract])) OR (Interleukin-10[Title/Abstract])) OR (Interleukin-11[Title/Abstract])) OR (Interleukin-12[Title/Abstract])) OR (Interleukin-12 Subunit p35[Title/Abstract])) OR (Interleukin-12 Subunit p40[Title/Abstract])) OR (Interleukin-13[Title/Abstract])) OR (Interleukin-15[Title/Abstract])) OR (Interleukin-16[Title/Abstract])) OR (Interleukin-17[Title/Abstract])) OR (Interleukin-18[Title/Abstract])) OR (Interleukin-2[Title/Abstract])) OR (Interleukin-23[Title/Abstract])) OR (Interleukin-23 Subunit p19[Title/Abstract])) OR (Interleukin-27[Title/Abstract])) OR (Interleukin-3[Title/Abstract])) OR (Interleukin-33[Title/Abstract])) OR (Interleukin-4[Title/Abstract])) OR (Interleukin-5[Title/Abstract])) OR (Interleukin-6[Title/Abstract])) OR (Interleukin-7[Title/Abstract])) OR (Interleukin-8[Title/Abstract])) OR (Interleukin-9[Title/Abstract])) OR (Cytokines[Title/Abstract])) OR (Chemokines[Title/Abstract])) OR (Growth Differentiation Factor 15[Title/Abstract])) OR (Tumor Necrosis Factor Superfamily Ligands[Title/Abstract])) OR (Tumor Necrosis Factor-alpha[Title/Abstract])) OR (umor Necrosis Factor alpha[Title/Abstract])) OR (Tumor Necrosis Factor Ligand Superfamily Member 2[Title/Abstract])) OR (TNF Receptor Ligands[Title/Abstract])) OR

	(TNFalpha[Title/Abstract])) OR (TNF-alpha[Title/Abstract])) OR (Interferons[Title/Abstract])) OR (Interferon-gamma[Title/Abstract])) OR (Interferon-alpha[Title/Abstract])) OR (Interferon Type I[Title/Abstract])) OR (Interferon-beta[Title/Abstract])) OR (Transforming Growth Factor beta[Title/Abstract])) OR (Transforming Growth Factor beta1[Title/Abstract])) OR (Transforming Growth Factor beta2[Title/Abstract])) OR (Transforming Growth Factor beta3[Title/Abstract])) OR (C-Reactive Protein[Title/Abstract]) AND (randomizedcontrolledtrial[Filter]))
Cochrane Central Register of Controlled Trials	renal insufficiency, chronic OR kidney disease OR renal disease OR kidney injury OR renal injury OR kidney insufficiency OR kidney dysfunction OR renal dysfunction OR kidney abnormal OR renal abnormal OR kidney damage OR renal damage OR kidney failure OR renal failure OR kidney complication OR renal complication OR kidney manifestation OR renal manifestation OR end-stage renal OR end-stage kidney OR endstage renal OR endstage kidney OR chronic kidney disease OR chronic renal disease OR Renal Replacement Therapy OR pre HD OR pre-HD OR HD OR hemoHD OR CKD

	Exercise OR Acute Exercise OR Isometric Exercise OR Aerobic Exercise OR Exercise Training OR Cool Down Exercise OR Cooldown Exercise OR Cooling-Down Exercise OR Cooling Down Exercise OR Warming-Down Exercise OR Warm Down Exercise OR Warm-Up Exercise OR Warm Up Exercise OR Warmup Exercise OR Warming Up Exercise OR Warming-Up Exercises OR Remedial Exercise OR Exercise Therapy OR Exercise Therapies OR Rehabilitation Exercise OR Physical Activity OR Physical activities OR Physical Exercise OR Gymnastics OR Calisthenics OR Movement OR Locomotor Activity OR Locomotor Activities OR Human Physical Conditioning OR Human Physical Training OR Circuit Based Exercise OR Circuit Training OR Endurance Training OR Physical Endurance OR Muscle Stretching Exercise OR Static Stretching OR Passive Stretching OR Resistance Training OR Strength Training OR Motor Activity OR Motor Activities OR Walking OR Ambulation<br>Inflammation OR Innate Inflammatory Response OR Systemic Inflammatory Response Syndrome OR Interleukin OR Cytokines OR Chemokines OR C Reactive Protein
LILACS	Kidney Failure, Chronic OR Renal Insufficiency, Chronic OR Renal Insufficiency OR Kidney Diseases OR Renal HD OR Hemodiafiltration OR HemoHD, Home OR Peritoneal HD OR

	Peritoneal HD, Continuous Ambulatory OR Renal Replacement Therapy OR Continuous Renal Replacement Therapy OR Intermittent Renal Replacement Therapy OR Hemofiltration OR Hemoperfusion OR Hybrid Renal Replacement Therapy OR Cardio-Renal Syndrome.<br>Exercise OR Cool-Down Exercise OR Gymnastics OR Muscle Stretching Exercises OR Physical Conditioning, Human OR Circuit-Based Exercise OR Endurance Training OR High-Intensity Interval Training OR Plyometric Exercise OR Resistance Training OR Exercise Therapy OR Preoperative Exercise OR Running OR Jogging OR Swimming OR Walking OR Stair Climbing OR Warm-Up Exercise OR Motor Activity OR Movement<br>Inflammation OR Interleukins OR Interleukin-1 OR Interleukin-1alpha OR Interleukin-1beta OR Interleukin-10 OR Interleukin-11 OR Interleukin-12 OR Interleukin-12 Subunit p35 OR Interleukin-12 Subunit p40 OR Interleukin-13 OR Interleukin-15 OR Interleukin-16 OR Interleukin-17 OR Interleukin-18 OR Interleukin-2 OR Interleukin-23 OR Interleukin-23 Subunit p19 OR Interleukin-27 OR Interleukin-3 OR Interleukin-33 OR Interleukin-4 OR Interleukin-5 OR Interleukin-6 OR Interleukin-7 OR Interleukin-8 OR Interleukin-9 OR Cytokines OR Chemokines OR Growth Differentiation Factor 15 OR Tumor Necrosis Factors

	OR Tumor Necrosis Factor-alpha OR Interferons OR Interferon-gamma OR Interferon-alpha OR Interferon Type I OR Interferon-beta OR Transforming Growth Factor beta OR Transforming Growth Factor beta1 OR Transforming Growth Factor beta2 OR Transforming Growth Factor beta3 OR C-Reactive Protein.

Table S2. Characteristics of included randomized controlled trials.

**Type of exercise**	**Frequency**	**Intensity**	**Inflammatory cytokines**	**Results**
Resistance and aerobic	3 times/week	Borg scale: 12	hs-CRP	↓hs-CRP
Resistance Training	3 times/week	Ankle weight: 0-5kg	IL-6, IL-10, TNF-α and hs-	↓CRP ↑IL-6
Resistance Training	3 times/week	OMNI-RES Scale:5-7	IL-6, IL-10, and TNF-α	↔IL-6 ↔IL-10
Aerobic training	3 times/week	65-75% of max Heart	CRP	↓CRP
Resistance	3 times/week	OMNI-RES	IL-10 and	↓TNF-α
Resistance and aerobic	2 times/week	Borg scale: 11-13.	hs-CRP	↔hs-CRP
Resistance and aerobic	3 times/week	50%–60% of VO2peak	IL-6 and hs-CRP.	↔IL-6 ↔hs-
Aerobic training	3 times/week	50%-60% of VO2peak	hs-CRP	↔hs-CRP
Resistance	3 times/week	60% of 1RM	hs-CRP	↔hs-

Table S2. Characteristics of included randomized controlled trials.

Country	Groups and age	Chronic kidney	Length of follow-up	Setting
Brazil	EX (n=76): 65±1.2 years CTL (n=74): 65±1.3 years	stages 2–4	16 weeks	Supervised location
China	EX (n=21): 59.0 (32.5-66.5) years [illegible]	HD	12 weeks	Intradialytic exercise
Brazil	MIEX= (n=16): 56.2±12.5 years	HD	12 weeks	Intradialytic exercise
Brazil	EX (n=15): 50±17.2 years CTL= (n=15): 58±15 years	HD	16 weeks	Intradialytic exercise
Brazil	EX (n=30): 66.0±4.0 years [illegible]	HD	12 weeks	Intradialyt[illegible]
Indonesia	AT (n=42): 49.78±11.65 years	HD	12 weeks	Intradialytic exercise
USA	EX (n=10): 57.5±11.5 years CTL (n=11): 52.5±10.6 years	Stages 2–4	48 weeks	Supervised location
USA	EX (n=25): 58.0±8.0 years CTL (n=21): 57.1±9.0 years	Stage 3	16 weeks	Supervised location
Brazil	EX (n=25): 45.7±15.2 years [illegible]	HD	12 weeks	Intradialyt[illegible]

Frequency	Intensity	Inflammatory cytokines	Results
3 times/week	Borg scale: 15-17	IL-6, IL-10 and TNF-α	↔IL-6 ↔IL-10
3 times/week	Borg scale: 12-15	hs-CRP and IL-[illegible]	↓hs-CRP [illegible]
3 times/week	80% of 1RM	CRP and IL-6	↓CRP [illegible]
3 times/week	25-85% of VO2 peak	CRP.	↔CRP.
3 times/week	Borg scale: 12-14	CRP and IL-6	↔CRP [illegible]
3 times/week	Borg scale: 15-17	CRP	↓CRP
3 times/week	40-60% of rest heart rate	CRP, TNF and IL-6.	↔CRP ↔TNF
3 times/week	50% of 1RM	CRP	↓CRP
3 times/week	60%–80% of VO2 peak	IL-6 and IL-10	↓IL-6 ↔IL-10

Authors
Barcellos et al. (2018)
Dong et al. (2019)
Lopes et al. (2019)
Silva et al. (2019)
Correa et al. [illegible]
Suhardjono et al. (2019)
Headley et al. (2012)
Headley et al. (2014)
Abreu et al. [illegible]

Table S2 (continued).

Groups and age	Chronic kidney	Length of follow-up	Setting	Type of exercise
EX (n=13): 60.2±15.2 years CTL (n=18): 66.3±13.5 years	HD	12 weeks	Intradialytic exercise	Resistance Training
EX (n=20): 62±8 years [illegible]	HD	12 weeks	Intradialytic [illegible]	Aerobic [illegible]
EX (n=14): 65±9 years [illegible]	Stages 1-4	12 weeks	Supervised [illegible]	Resistance [illegible]
EX (n=7) CTL (n = 4):	Stages 2-4	24 weeks	Home based	Aerobic training
EX (n=8): 60.8±3.2 years [illegible]	HD	16 weeks	Intradialyt [illegible]	Aerobic [illegible]
EX (n=24): 60.0±15.3 years [illegible]	HD	12 weeks	Intradialyt [illegible]	Resistance [illegible]
EX (n=5): 46±13 years CTL (n=6): 52,2±17 years	HD	16 weeks	Intradialyt ic exercise	Resistance and aerobic
RT (n=14): 48.9±10.1 years CTL (n=14): 51.9±11.6 years	HD	10 weeks	Intradialyt ic exercise	Resistance Training
AEG/Usual Diet (n=27): 58.0(48.5-62.0) years	Stages 3-4	16 weeks	Supervised location	Aerobic training

Intensity	Inflammatory cytokines	Results
50-65% of 1RM. Borg scale: 5-6	CRP	↔ CRP
Borg scale: 11-12	CRP	↔ CRP
30-70% of 1-RM	TNF-α, IL-6 and IL-10	↓TNF-α ↓IL-6
OMNI Scale: 5-8	TNF-α, IL-6, IL-10	↓TNF-α ↓IL-6
Borg scale: 12-17 60% of 3RM	hs-CRP	↓hs-CRP
20km/h	IL-6	↓IL-6
Borg scale: 2-7	IL-6, IL-10 and TNF-α	↓IL-6 ↓TNF-α
Borg scale: 12–14	IL-6, IL10 and CRP	"↔CRP ↔IL-6
Borg scale: 12–14	IL-6, IL-10 and TNF-α	↔TNF-α ↔IL-6
Borg scale: 12–14	CRP, IL-6, IL-10 and TNF-α	↔CRP ↔TNF-α

Authors	Country
Cheema et al. (2011)	Australia
Liao et al. (2016)	Taiwan.
Castaneda et al.	USA
Leehey et al. (2009)	USA
Wilund et al. (2010)	USA
Cheema et al. (2007)	Australia
Oliveros et al. (2011)	Chile
Pellizzaro et al.	Brazil
Alp Ikizler et al.	USA

Table S2 (continued).

Chronic kidney	Length of follow-up	Setting	Type of exercise	Frequency
HD	16 weeks	Interdialytic exercise	Resistance and aerobic	4 times/week
HD	96 weeks	Intradialytic [illegible]	Resistance [illegible]	3 times/week
Stage 2	24 weeks	Supervised location	Resistance Training	3 times/week
HD	24 weeks	Interdialytic exercise	Resistance Training	3 times/week
HD	8 weeks	Intradialytic exercise	Resistance and aerobic	3 times/week
HD	18 weeks	Interdialytic exercise	Aerobic training	6 times/week
HD	12 weeks	Intradialytic exercise	Aerobic training	3 times/week
Stage 4-5	24 weeks	Home based	Aerobic training	5 times/week
HD	24 weeks	Intradialytic exercise	Aerobic training	3 times/week
HD	24 weeks	Intradialytic exercise	Aerobic training	3 times/week

Results
↓CRP
↔CRP<br>↔TNF-α

Authors	Country	Groups and age
Frih et al. (2017)	Tunisia	EX (n=21): 64.2±3.4<br>CTL (n=20): 65.2±3.1
Cheng et al. [illegible]	China	EX (n=67): 54.64±12.55<br>[illegible]
Correa et al. (2021)	Brazil	RT (n=35): 58±6 years<br>CTL (n=35): 58±5 years
Moura et al. (2020)	Brazil	EX (n=81): 67.3±3.2 years<br>CTL (n=76): 66.3±3.9 years
Afshar et al. (2010)	Iran	AT (n=7): 50.7±21.06 years<br>RT (n=7): 51±16.4 years
Zhao et al. (2016)	China	CTL (n=56): 54.1(42.3–68.7) years
Cruz et al. (2018)	Brazil	EX (n=15): 43.5±14.4 years<br>CTL (n=15): 39.9±13.5 years
Viana et al. (2014)	England	"EX (n=13): 61±8 years<br>CTL (n=11): 56±16 years"
Highton et al. (2021)	England	EX (n=20): 51.4±18.1 years<br>CTL (n=20): 56.8±14.0 years
March et al. (2022)	England	EX (n=46): 51.4±18.1 years<br>CTL (n=46): 56.8±14.0 years

**Length of follow-up**	**Setting**	**Type of exercise**	**Frequency**	**Intensity**	**Inflammatory cytokines**
8 weeks	Intradialytic exercise	Aerobic training	3 times/week	Borg scale: 12–15	CRP
21 weeks	Intradialytic exercise	Resistance and aerobic	3 times/week	50% of VO2 peak 70% of 1RM	CRP, IL-6 and TNF-α

**Authors**	**Country**	**Groups and age**	**Chronic kidney**
Afshar et al. (2011)	Iran	EX (n=14): 21.06±50.71 years	HD
Kopple et al. (2007)	USA	EX (n=12): 42.7±3.8 years<br>CTL (n=14): 41.3±3.3 years	HD

Table S3. PEDro scale.

**Follow-Up**	**Intention-to-Treat Analysis**	**Between-Group Comparisons**	**Point Measures and Variability**	**PEDro Score**
0	0	1	1	**4**
1	0	1	1	**5**
0	0	1	1	**3**
1	0	1	1	**4**
0	0	1	1	**3**
1	0	1	1	**4**
0	0	1	1	**4**
1	0	1	1	**4**
0	0	1	1	**3**

Table S3. PEDro scale.

**Random Allocation**	**Concealed Allocation**	**Groups Similar At Baseline**	**Blind Subject**	**Blind Therapist**	**Blind Assessor**
1	0	1	0	0	0
1	0	1	0	0	0
1	0	0	0	0	0
1	0	0	0	0	0
1	0	0	0	0	0
1	0	0	0	0	0
1	0	1	0	0	0
1	0	0	0	0	0
1	0	0	0	0	0

**Authors**	**Eligibility Criteria**
Barcellos et al. (2018)	1
Dong et al. (2019)	1
Lopes et al. (2019)	1
Silva et al. (2019)	1
Correa et al. (2020)	1
Suhardjono et al. (2019)	1
Headley et al. (2012)	1
Headley et al. (2014)	1
Abreu et al. (2017)	1

**Follow-Up**	**Intention-to-Treat Analysis**	**Between-Group Comparisons**	**Point Measures and Variability**	**PEDro Score**
0	0	1	1	**4**
1	0	1	1	**4**
1	0	1	1	**5**
0	0	1	1	**3**
1	0	1	1	**5**
1	1	1	1	**6**
0	0	1	1	**3**
1	1	1	1	**6**
1	1	1	1	**6**

Table S3 (continued).

**Random Allocation**	**Concealed Allocation**	**Groups Similar At Baseline**	**Blind Subject**	**Blind Therapist**	**Blind Assessor**
1	0	1	0	0	0
1	0	0	0	0	0
1	0	1	0	0	0
1	0	0	0	0	0
1	0	1	0	0	0
1	0	1	0	0	0
1	0	0	0	0	0
1	0	1	0	0	0
1	0	1	0	0	0

**Authors**	**Eligibility Criteria**
Cheema et al. (2011)	1
Liao et al. (2016)	1
Castaneda et al. (2004)	1
Leehey et al. (2009)	1
Wilund et al. (2010)	1
Cheema et al. (2007)	1
Oliveros et al. (2011)	1
Pellizzaro et al. (2013)	1
Alp Ikizler et al. (2018)	1

**Follow-Up**	**Intention-to-Treat Analysis**	**Between-Group Comparisons**	**Point Measures and Variability**	**PEDro Score**
0	0	1	1	**4**
0	0	1	1	**4**
0	0	1	1	**4**
0	0	1	1	**4**
1	1	1	1	**6**
1	1	1	1	**6**
1	1	1	1	**5**
0	0	1	1	**3**
0	1	1	1	**5**
0	1	1	1	**5**

Table S3 (continued).

**Random Allocation**	**Concealed Allocation**	**Groups Similar At Baseline**	**Blind Subject**	**Blind Therapist**	**Blind Assessor**
1	0	1	0	0	0
1	0	1	0	0	0
1	0	1	0	0	0
1	0	1	0	0	0
1	0	1	0	0	0
1	0	1	0	0	0
1	0	0	0	0	0
0	0	1	0	0	0
1	0	1	0	0	0
1	0	1	0	0	0

**Authors**	**Eligibility Criteria**
Frih et al.	1
Cheng et al. (2019)	1
Correa et al. (2021)	1
Moura et al. (2020)	1
Afshar et al. (2010)	1
Zhao et al. (2017)	1
Cruz et al. (2018)	1
Viana et al. (2014)	1
Highton et al. (2021)	1
March et al. (2022)	1

**Follow-Up**	**Intention-to-Treat Analysis**	**Between-Group**	**Point Measures and**	**PEDro**
1	1	1	1	**6**
0	1	1	1	**5**

Table S3

**Random Allocatio**	**Concealed Allocation**	**Groups Similar At**	**Blind Subjec**	**Blind Therapist**	**Blind Assessor**
1	0	1	0	0	0
1	0	1	0	0	0

Authors	Eligibility
Afshar et al. (2011)	1
Kopple et al. (2007)	1

Table S4. GRADE analysis in comparison the exercise intervention and usual care control groups.

Certainty assessment							№ of patients		Effect		Certainty	Importance
№ of studies	Study design	Risk of bias	Inconsistency	Indirectness	Imprecision	Other considerations	Exercise	Usual care	Relative (95% CI)	Absolute (95% CI)		
**CRP (follow-up: mean 21 weeks; assessed with: Blood test; Scale from: -0.44 to 0.03)**												

17[17,18,19,21,28,30,32,33,34,35,36,37,38,39,40,41,43,45,47,48,49,50]	randomised trials	serious[1,8,10,12,13,14,16,17,18,19,a,b]	not serious[c,d]	serious[e,f,g]	serious[h,i]	none	376	386	-	mean **0.2 lower** (0.44 lower to 0.03 higher)	⊕◯◯◯ Very low	IMPORTANT
**TNF (follow-up: mean 16 weeks; assessed with: blood test; Scale from: -6.02 to -2.55)**												
7[1,4,19,20,22,23,24,25,26,27]	randomised trials	serious[1,19,22,23,24,25,26,27,a,b]	not serious[c,d]	not serious[j,k,l]	not serious[m,n]	none	203	197	-	mean **4.28 lower** (6.02	⊕⊕⊕◯	IMPORTANT

Certainty assessment							№ of patients		Effect		Certainty	Importance
№ of studies	Study design	Risk of bias	Inconsistency	Indirectness	Imprecision	Other considerations	Exercise	Usual care	Relative (95 % CI)	Absolute (95% CI)		
										lower to 2.55 lower)	Moderate	

IL-6 (follow-up: mean 18 weeks; assessed with: Blood test; Scale from: -1.31 to -0.16)

13[4,8,11,13,16,18,19,20,22,23,25,26,27,28,29]	randomised trials	Serious[18,21,28,36,37,38,43,45,49,50,a,b]	not serious[c,d]	serious[e,f,g]	not serious[m,o]	none	265	268	-	mean **0.73 lower** (1.31 lower to 0.16 lower)	⊕⊕◯◯ Low	IMPORTANT
**IL-10 (follow-up: mean 16 weeks; assessed with: Blood test; Scale from: 0.29 to 2.51)**												
8[4,13,14,20,21,29,30,31,42,46]	randomised trials	Serious[4,13,14,20,21,29,31,42,45,46,a,b]	serious[d,p]	serious[e,f,g]	not serious[m,o]	none	238	235	-	mean **1.4 higher**	⊕◯◯◯	IMPORTANT

Certainty assessment							№ of patients		Effect		Certainty	Importance
№ of studies	Study design	Risk of bias	Inconsistency	Indirectness	Imprecision	Other considerations	Exercise	Usual care	Relative (95% CI)	Absolute (95% CI)		
										(0.29 higher to 2.51 higher)	Very low	

Explanations

a. High dropout

b. Subject allocation was not blind

c. $i^2 < 75\%$

d. Similar length of intervention

e. Different CKD stages

f. Different types of training

g. Different setting

h. High standard deviation

i. Different results

j. Similar types of exercise

k. Similar setting

l. Similar CKD stages

m. Similar results

n. Low standard deviation

o. Similar standard deviation

p. $i^2 > 75\%$

+ means very low; ++ low; +++ moderate; and ++++ high certainty of evidence

Table S5. GRADE analysis in comparison the aerobic exercise intervention and usual care control groups.

Certainty assessment							№ of patients		Effect		Certainty	Importance
№ of studies	Study design	Risk of bias	Inconsistency	Indirectness	Imprecision	Other considerations	Aerobic interventions	Usual care	Relative (95% CI)	Absolute (95% CI)		
**CRP (follow-up: mean 15 weeks; assessed with: Blood test; Scale from: -0.78 to 0.35)**												

Certainty assessment							№ of patients		Effect		Certainty	Importance
№ of studies	Study design	Risk of bias	Inconsistency	Indirectness	Imprecision	Other considerations	Aerobic interventions	Usual care	Relative (95% CI)	Absolute (95% CI)		
8[19,21,32,34,36,37,38,43,45,48]	randomised trials	serious[21,34,36,38,43,45,a]	not serious[b,c]	serious[d,e]	serious[f]	none	124	115	-	mean **0.22 lower** (0.78 lower to 0.35 lower)	⨁◯◯◯ Very low	

IL-6 (follow-up: mean 14 weeks; assessed with: Blood test; Scale from: -1.67 to 0.33)

Certainty assessment							№ of patients		Effect		Certainty	Importance
№ of studies	Study design	Risk of bias	Inconsistency	Indirectness	Imprecision	Other considerations	Aerobic interventions	Usual care	Relative (95% CI)	Absolute (95% CI)		
5[14,21,29,36,38,45,46]	randomised trials	Serious[14,21,29,36,38,45,46,a]	not serious[b,c]	not serious	serious[g]	none	70	70	-	mean **0.67 lower** (1.67 lower to 0.33 higher)	⊕⊕◯◯ Low	

Certainty assessment							№ of patients		Effect		Certainty	Importance
№ of studies	Study design	Risk of bias	Inconsistency	Indirectness	Imprecision	Other considerations	Aerobic interventions	Usual care	Relative (95% CI)	Absolute (95% CI)		
**IL-10 (follow-up: mean 14 weeks; assessed with: Blood test; Scale from: -1.14 to 2.64)**												

Certainty assessment							№ of patients		Effect		Certainty	Importance
№ of studies	Study design	Risk of bias	Inconsistency	Indirectness	Imprecision	Other considerations	Aerobic interventions	Usual care	Relative (95% CI)	Absolute (95% CI)		
3[14,21,29,45,46]	randomised trials	Serious[21,a]	not serious[b,c]	very serious[d,e,g,h]	very serious[g,i]	none	42	41	-	mean **0.75 higher** (1.14 lower to 2.64 higher)	⊕◯◯◯ Very low	

Certainty assessment							№ of patients		Effect		Certainty	Importance
№ of studies	Study design	Risk of bias	Inconsistency	Indirectness	Imprecision	Other considerations	Aerobic interventions	Usual care	Relative (95% CI)	Absolute (95% CI)		
**TNF (follow-up: mean 12 weeks; assessed with: Blood test; Scale from: -5.69 to -0.99)**												
1[14,45,46]	randomised trials	serious[a]	not serious	not serious	very serious[g]	none	15	15	-	mean **3.34 lower** (5.69 lower to 0.99 lower)	⊕◯◯◯ Very low	

CI: confidence interva

Explanations

a. Subject allocation was not blind

b. $I^2 < 75\%$

c. Similar length of intervention

d. Different setting

e. Different CKD treatment

f. High standard deviation

g. Low sample size

h. Different CKD stages

i. Different results

Table S6. GRADE analysis in comparison the resistance exercise intervention and usual care control groups.:

Certainty assessment							№ of patients		Effect		Certainty	Importance
№ of studies	Study design	Risk of bias	Inconsistency	Indirectness	Imprecision	Other considerations	Resistance interventions	Usual care control	Relative (95% CI)	Absolute (95% CI)		
**CRP (follow up: mean 23 weeks; assessed with: Blood test; Scale from: -0.32 to -0.04)**												

Certainty assessment							№ of patients		Effect		Certainty	Importance
№ of studies	Study design	Risk of bias	Inconsistency	Indirectness	Imprecision	Other considerations	Resistance interventions	Usual care control	Relative (95% CI)	Absolute (95% CI)		
7 [28,30,35,39,41,43,49]	randomised trials	serious [28,35,43,49,a,b]	not serious [c,d]	not serious	serious [e,f]	none	121	148	-	mean **0.31 lower** (0.73 lower to 0.11 lower)	⊕⊕◯◯ LOW	IMPORTANT

IL-6 (follow up: mean 16 weeks; assessed with: Blood test; Scale from: -0.64 to -0.14)

Certainty assessment							№ of patients		Effect		Certainty	Importance
**№ of studies**	**Study design**	**Risk of bias**	**Inconsistency**	**Indirectness**	**Imprecision**	**Other considerations**	**Resistance interventions**	**Usual care control**	**Relative (95% CI)**	**Absolute (95% CI)**		
6 [4,31,30,,42,49]	randomised trials	serious [4,31,42,a,b]	not serious [c,d]	serious [f,g]	serious [e,f,h]	none	180	181	-	mean **1.02 lower** (2.08 lower to 0.03 higher)	⨁◯◯◯ VERY LOW	IMPORTANT

IL-10 (follow up: mean 16 weeks; assessed with: Blood test; Scale from: 0.32 to 0.78)

Certainty assessment							№ of patients		Effect		Certainty	Importance
**№ of studies**	**Study design**	**Risk of bias**	**Inconsistency**	**Indirectness**	**Imprecision**	**Other considerations**	**Resistance interventions**	**Usual care control**	**Relative (95% CI)**	**Absolute (95% CI)**		
6 [4,13,20,30,31,42]	randomised trials	serious [4,13,20,31,42,a,b]	very serious [e,h]	not serious [i,j,k]	serious [e]	none	196	194	-	mean **1.65 higher** (0.26 higher to 3.04 higher)	⨁◯◯◯ VERY LOW	IMPORTANT

TNF (follow up: mean 17 weeks; assessed with: Blood test; Scale from: -6.44 to -4.06)

Certainty assessment							№ of patients		Effect		Certainty	Importance
**№ of studies**	**Study design**	**Risk of bias**	**Inconsistency**	**Indirectness**	**Imprecision**	**Other considerations**	**Resistance interventions**	**Usual care control**	**Relative (95% CI)**	**Absolute (95% CI)**		
5 [4,20,30,31,42]	randomised trials	serious [4,20,31,42,a,b]	not serious [c,d]	not serious [i,j,k]	not serious [c,l]	none	183	176	-	mean **4.44 lower** (6.57 lower to 2.31 lower)	⨁⨁⨁◯ MODERATE	IMPORTANT

CI: Confidence interval

Explanations

a. High dropout

b. Allocation was not blind

c. $I^2 < 75\%$

d. Similar length of intervention

e. High standard deviation

f. Different results

g. Different setting

h. $I^2 > 75\%$

i. Similar type of exercise

j. Similar setting

k. Similar CKD stage

l. Low standard deviation

Table S7. GRADE analysis in comparison the combined exercise intervention and usual care control groups.

Certainty assessment							№ of patients		Effect		Certainty	Importance
№ of studies	Study design	Risk of bias	Inconsistency	Indirectness	Imprecision	Other considerations	Combined interventions	Usual care	Relative (95% CI)	Absolute (95% CI)		
**CRP (follow-up: mean 22 weeks; assessed with: Blood test; Scale from: -0.73 to 0.77)**												

Certainty assessment							№ of patients		Effect		Certainty	Importance
№ of studies	Study design	Risk of bias	Inconsistency	Indirectness	Imprecision	Other considerations	Combined interventions	Usual care	Relative (95% CI)	Absolute (95% CI)		
5[18,32,33,40,47,50]	randomised trials	Serious[18,28,33,50, a,b]	not serious[c,d]	serious[e,f]	serious[g,h]	none	131	123	-	mean **0.02 higher** (0.73 lower to 0.77 higher)	⨁◯◯◯ Very low	

IL-6 (follow-up: mean 32 weeks; assessed with: Blood test; Scale from: -2.51 to 0.76)

Certainty assessment							№ of patients		Effect		Certainty	Importance
№ of studies	Study design	Risk of bias	Inconsistency	Indirectness	Imprecision	Other considerations	Combined interventions	Usual care	Relative (95% CI)	Absolute (95% CI)		
2[33,47,50]	randomised trials	Serious[33,50],[a,b]	not serious[c]	serious[e,f]	serious[g,i]	none	15	17	-	mean **0.88 lower** (2.51 lower to 0.76 higher)	⨁◯◯◯ Very low	

TNF (follow-up: mean 16 weeks; assessed with: Blood test; Scale from: -21.87 to 21.03)

Certainty assessment							№ of patients		Effect		Certainty	Importance
№ of studies	Study design	Risk of bias	Inconsistency	Indirectness	Imprecision	Other considerations	Combined interventions	Usual care	Relative (95% CI)	Absolute (95% CI)		
1[47,50]	randomised trials	Serious[50,b]	not serious	not serious	very serious[g,i]	none	5	6	-	mean **0.42 lower** (21.87 lower to 21.03 higher)	⨁◯◯◯ Very low	

CI: confidence interval

Explanations

a. High dropout

b. Subjects allocation was not blind

c. $I^2 < 75\%$

d. Similar interventions time

e. Different CKD treatment

f. Different setting

g. High standard deviation

h. Different results

i. Low sample size

Table S8. Fixed effects model meta-analyses performed in the review showing the effect of exercise in CRP, IL-6, IL-10 and TNF-α levels compared to control group.

Exercise intervention	Studies	Individuals	Std. Mean difference (95% CI)	Heterogeneity (i2, %)
**Pooled exercise interventions**				
CRP	21	932	-0.22 (-0.35 to -0.09)	44
IL-6	16	715	-0.31 (-0.46 to -0.16)	44
IL-10	11	629	0.45 (0.29 to 0.61)	87
TNF-α	11	558	-0.58 (-0.76 to -0.41)	74
**Aerobic interventions**				
CRP	10	383	-0.25 (-0.46 to -0.04)	65
IL-6	7	296	-0.17 (-0.40 to 0.06)	28
IL-10	5	239	0.05 (-0.21 to 0.31)	78
TNF-α	3	122	-0.29 (-0.61 to 0.02)	55
**Resistance interventions**				
CRP	7	269	-0.38 (-0.63 to -0.12)	27
IL-6	6	361	-0.45 (-0.66 to -0.24)	66
IL-10	6	390	0.72 (0.51 to 0.93)	89
TNF-α	5	359	-0.81 (-1.03 to -0.59)	77
**Combined interventions**				
CRP	6	280	-0.05 (-0.29 to 0.18)	0
IL-6	3	58	-0.16 (-0.68 to 0.36)	0
IL-10	0	0	-	-
TNF-α	2	37	0.16 (-0.49 to 0.51)	0

IL = interleukin; TNF-α = Tumor necrosis factor alpha; CRP = C reactive protein; CI = confidence interval; SMD: standard mean difference.

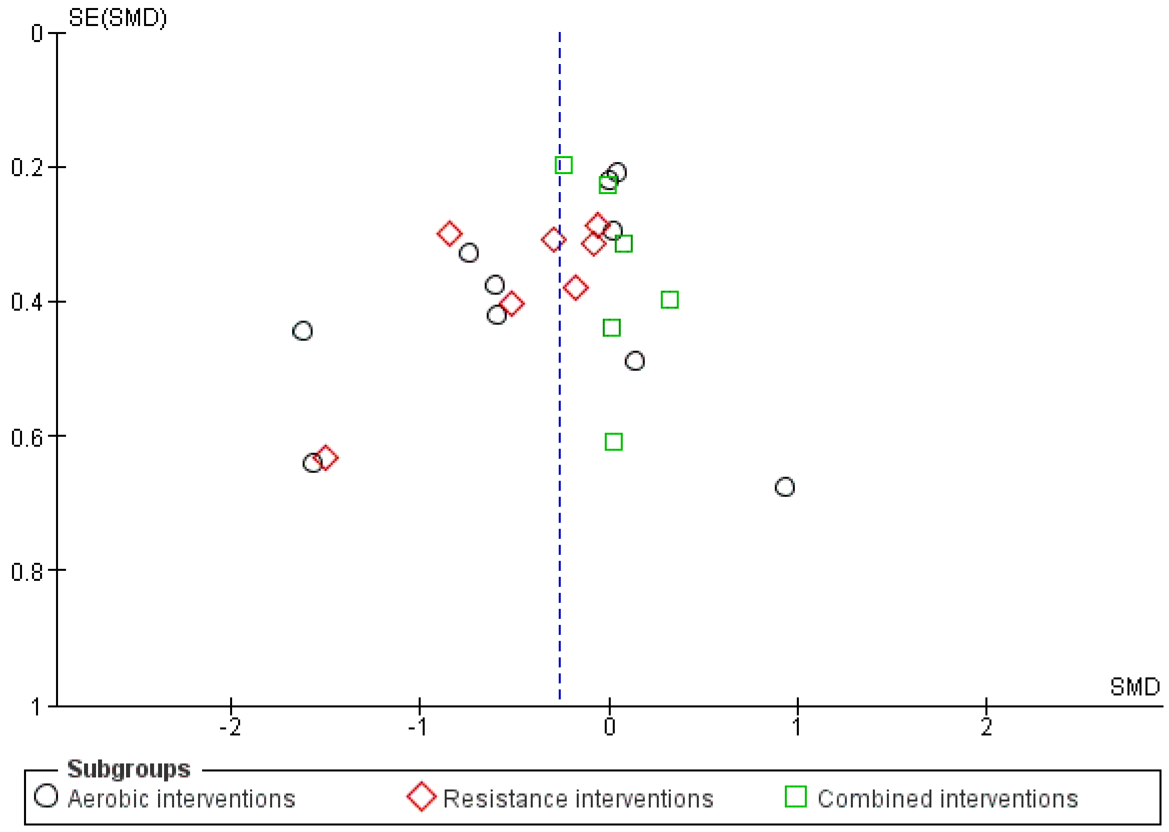

Figure S1. Funnel plot of the difference in C-Reactive Protein (CRP) between exercise intervention types.

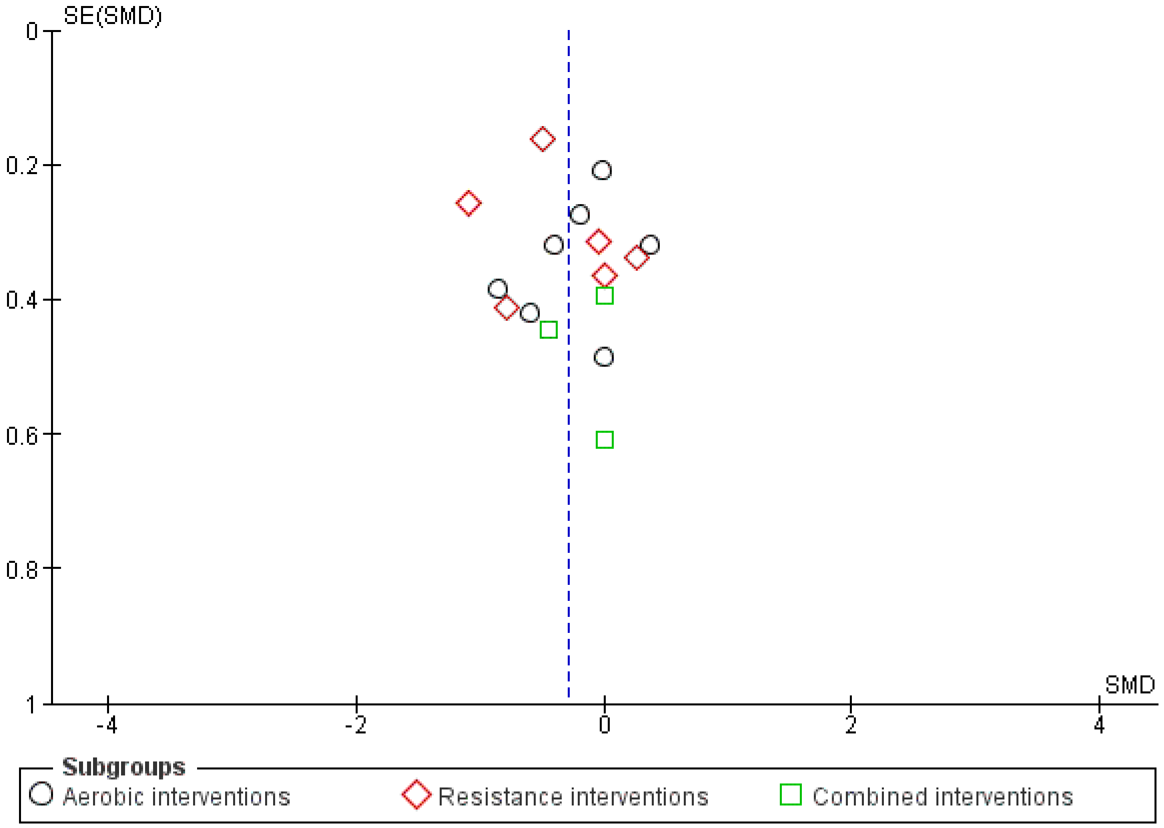

Figure S2. Funnel plot of the difference in Interleukin-6 (IL-6) between exercise intervention types.

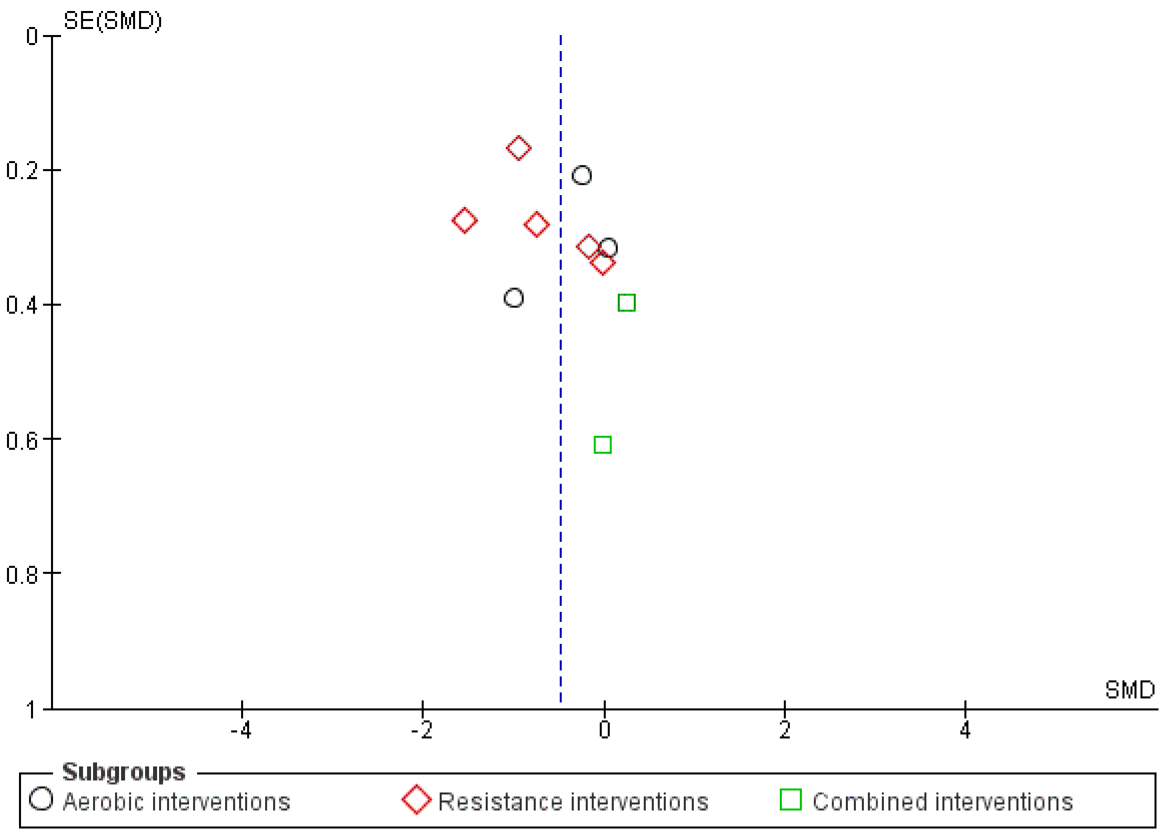

Figure S3. Funnel plot of the difference in Interleukin-10 (IL-10) between exercise intervention types.

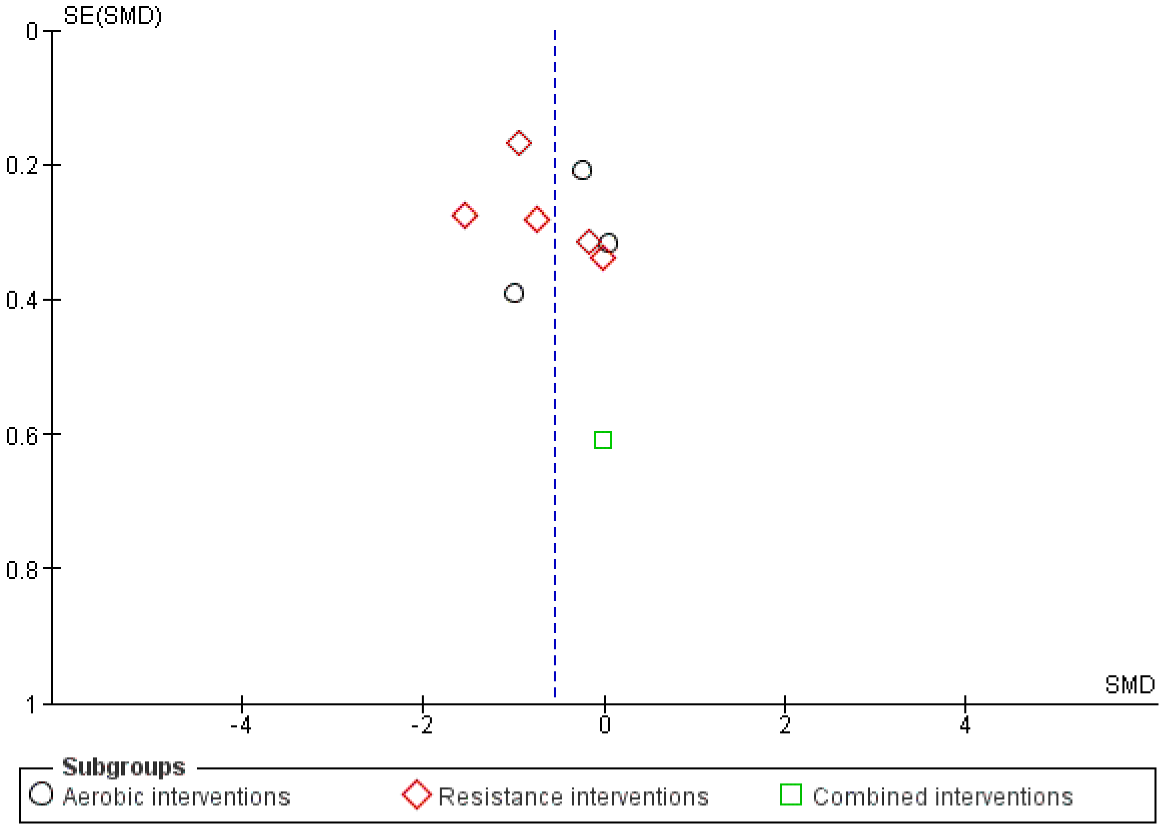

Figure S4. Funnel plot of the difference in Tumour Necrosis Factor-alpha (TNF-α) between exercise intervention types.

References

4. Moura, S.R.G.; Corrêa, H.L.; Neves, R.V.P.; Santos, C.A.R.; Neto, L.S.S.; Silva, V.L.; Souza, M.K.; Deus, L.A.; Reis, A.L.; Simões, H.G.; et al. Effects of resistance training on hepcidin levels and iron bioavailability in older individuals with end-stage renal disease: A randomized controlled trial. *Exp. Gerontol*. **2020**, *139*, 111017.

13. Cheema, B.S.B.; Abas, H.; Smith, B.C.F.; O'sullivan, A.J.; Chan, M.; Patwardhan, A.; Kelly, J.; Gillin, A.; Pang, G.; Lloyd, B.; et al. Effect of resistance training during hemodialysis on circulating cytokines: A randomized controlled trial. *Eur. J. Appl. Physiol*. **2011**, *111*, 1437–1445.

14. Cruz LG da Zanetti, H.R.; Andaki, A.C.R.; Mota GR da Barbosa Neto, O.; Mendes, E.L. Intradialytic aerobic training improves inflammatory markers in patients with chronic kidney disease: A randomized clinical trial. *Mot. Rev. Educ. Física*. **2018**, *24*, 1–5.

18. Barcellos, F.C.; Del Vecchio, F.B.; Reges, A.; Mielke, G.; Santos, I.S.; Umpierre, D.; Bohlke, M.; Hallal, P. Exercise in patients with hypertension and chronic kidney disease: A randomized controlled trial. *J. Hum. Hypertens.* **2018**, *32*, 397–407.

19. Oliveira ESilva, V.R.; Stringuetta Belik, F.; Hueb, J.C.; de Souza Gonçalves, R.; Costa Teixeira Caramori, J.; Perez Vogt, B.; Barretti, P.; Zanati Bazan, S.G.; De Stefano,

G.M.M.F.; Martin, L.C.; et al. Aerobic Exercise Training and Nontraditional Cardiovascular Risk Factors in Hemodialysis Patients: Results from a Prospective Randomized Trial. *Cardiorenal Med.* **2019**, *9*, 391–399.

20. Corrêa, H.L.; Moura, S.R.G.; Neves, R.V.P.; Tzanno-Martins, C.; Souza, M.K.; Haro, A.S.; Costa, F.; Silva, J.A.B.; Stone, W.; Honorato, F.S.; et al. Resistance training improves sleep quality, redox balance and inflammatory profile in maintenance hemodialysis patients: A randomized controlled trial. *Sci. Rep.* **2020**, *10*, 11708. https://doi.org/10.1038/s41598-020-68602-1

21. Viana, J.L.; Kosmadakis, G.C.; Watson, E.L.; Bevington, A.; Feehally, J.; Bishop, N.C.; Smith, A.C. Evidence for Anti-Inflammatory Effects of Exercise in CKD. *J. Am. Soc. Nephrol.* **2014**, *25*, 2121–2130.

28. Pellizzaro, C.O.; Thomé, F.S.; Veronese, F.V. Effect of peripheral and respiratory muscle training on the functional capacity of hemodialysis patients. *Ren. Fail*. **2013**, *35*, 189–197.

29. Ikizler, T.A.; Robinson-Cohen, C.; Ellis, C.; Headley, S.A.; Tuttle, K.; Wood, R.J.; Evans, E.E.; Milch, C.M.; Moody, K.A.; Germain, M.; et al. Metabolic Effects of Diet and Exercise in Patients with Moderate to Severe CKD: A Randomized Clinical Trial. *J. Am. Soc. Nephrol*. **2018**, *29*, 250–259.

30. Dong, Z.J.; Zhang, H.L.; Yin, L.X. Effects of intradialytic resistance exercise on systemic inflammation in maintenance hemodialysis patients with sarcopenia: A randomized controlled trial. *Int. Urol. Nephrol*. **2019**, *51*, 1415–1424. https://doi.org/10.1007/s11255-019-02200-7

31. Lopes, L.C.C.; Mota, J.F.; Prestes, J.; Schincaglia, R.M.; Silva, D.M.; Queiroz, N.P.; Freitas, A.T.V.D.S.; Lira, F.D.S.; Peixoto, M.D.R.G. Intradialytic Resistance Training Improves Functional Capacity and Lean Mass Gain in Individuals on Hemodialysis: A Randomized Pilot Trial. *Arch Phys. Med. Rehabil.* **2019**, *100*, 2151–2158.

32. Suhardjono Umami, V.; Tedjasukmana, D.; Setiati, S. The effect of intradialytic exercise twice a week on the physical capacity, inflammation, and nutritional status of dialysis patients: A randomized controlled trial. *Hemodial. Int.* **2019**, *23*, 486–493.

33. 33. Headley, S.; Germain, M.; Milch, C.; Pescatello, L.; Coughlin, M.A.; Nindl, B.C.; Cornelius, A.; Sullivan, S.; Gregory, S.; Wood, R. Exercise Training Improves HR Responses and V˙O2peak in Predialysis Kidney Patients. *Med. Sci. Sport Exerc.* **2012**, *44*, 2392–2399.

34. Headley, S.; Germain, M.; Wood, R.; Joubert, J.; Milch, C.; Evans, E.; Poindexter, A.; Cornelius, A.; Brewer, B.; Pescatello, L.S.; et al. Short-term aerobic exercise and vascular function in CKD stage 3: A randomized controlled trial. *Am. J. Kidney Dis.* **2014**, *64*, 222–229.

35. Abreu, C.; Cardozo, L.; Stockler-Pinto, M.; Esgalhado, M.; Barboza, J.; Frauches, R.; Mafra, D. Does resistance exercise performed during dialysis modulate Nrf2 and NF-κB in patients with chronic kidney disease? *Life Sci.* **2017**, *188*, 192–197. http://dx.doi.org/10.1016/j.lfs.2017.09.007

36. Liao, M.-T.; Liu, W.-C.; Lin, F.-H.; Huang, C.-F.; Chen, S.-Y.; Liu, C.-C.; Lin, S.-H.; Lu, K.-C.; Wu, C.-C. Intradialytic aerobic cycling exercise alleviates inflammation and improves endothelial progenitor cell count and bone density in hemodialysis patients. *Medicine* **2016**, *95*, e4134.

37. Leehey, D.J.; Moinuddin, I.; Bast, J.P.; Qureshi, S.; Jelinek, C.S.; Cooper, C.; Edwards, L.C.; Smith, B.M.; Collins, E.G. Aerobic exercise in obese diabetic patients with chronic kidney disease: A randomized and controlled pilot study. *Cardiovasc. Diabetol.* **2009**, *8*, 62–68.

38. Wilund, K.R.; Tomayko, E.J.; Wu, P.-T.; Chung, H.R.; Vallurupalli, S.; Lakshminarayanan, B.; Fernhall, B. Intradialytic exercise training reduces oxidative stress and epicardial fat: A pilot study. *Nephrol. Dial. Transplant*. **2010**, *25*, 2695–2701.

39. Cheema, B.; Abas, H.; Smith, B.; O'Sullivan, A.; Chan, M.; Patwardhan, A.; Kelly, J.; Gillin, A.; Pang, G.; Lloyd, B.; et al. Progressive exercise for anabolism in kidney disease (PEAK): A randomized, controlled trial of resistance training during hemodialysis. *J. Am. Soc. Nephrol*. **2007**, *18*, 1594–1601.

40. Frih, B.; Jaafar, H.; Mkacher, W.; Salah ZBen Hammami, M.; Frih, A. The effect of interdialytic combined resistance and aerobic exercise training on health related outcomes in chronic hemodialysis patients: The Tunisian randomized controlled study. *Front Physiol.* **2017**, *8*, 288.

41. Cheng, Y.J.; Zhao, X.J.; Zeng, W.; Xu, M.C.; Ma, Y.C.; Wang, M. Effect of Intradialytic Exercise on Physical Performance and Cardiovascular Risk Factors in Patients Receiving Maintenance Hemodialysis: A Pilot and Feasibility Study. *Blood Purif.* **2020**, *49*, 409–418.

42. Corrêa, H.L.; Neves, R.V.P.; Deus, L.A.; Souza, M.K.; Haro, A.S.; Costa, F.; Silva, V.L.; Santos, C.A.R.; Moraes, M.R.; Simões, H.G.; et al. Blood Flow Restriction Training Blunts Chronic Kidney Disease Progression in Humans. *Med. Sci. Sport Exerc*. **2021**, *53*, 249–257.

43. Afshar, R.; Shegarfy, L.; Shavandi, N.; Sanavi, S. Effects of aerobic exercise and resistance training on lipid profiles and inflammation status in patients on maintenance hemodialysis. *Indian J. Nephrol*. **2010**, *20*, 185–189.

45. March, D.S.; Lai, K.-B.; Neal, T.; Graham-Brown, M.P.M.; Highton, P.J.; Churchward, D.R.; Young, H.M.L.; Dungey, M.; Stensel, D.J.; Smith, A.C.; et al. Circulating endotoxin and inflammation: Associations with fitness, physical activity and the effect of a 6-month programme of cycling exercise during haemodialysis. *Nephrol. Dial. Transplant*. **2022**, *37*, 366–374.

46. Highton, P.J.; March, D.S.; Churchward, D.R.; Grantham, C.E.; Young, H.M.L.; Graham-Brown, M.P.M.; Estruel, S.; Martin, N.; Brunskill, N.J.; Smith, A.C.; et al. Intradialytic cycling does not exacerbate microparticles or circulating markers of systemic inflammation in haemodialysis patients. *Eur. J. Appl. Physiol.* **2021**, *122*, 599–609. https://doi.org/10.1007/s00421-021-04846-7

47. Kopple, J.D.; Wang, H.; Casaburi, R.; Fournier, M.; Lewis, M.I.; Taylor, W.; Storer, T.W. Exercise in maintenance hemodialysis patients induces transcriptional changes in genes favoring anabolic muscle. *J. Am. Soc. Nephrol*. **2007**, *18*, 2975–2986.

48. Afshar, R.; Emany, A.; Saremi, A.; Shavandi, N.; Sanavi, S. Effects of intradialytic aerobic training on sleep quality in hemodialysis patients. *Iran J. Kidney Dis*. **2011**, *5*, 119–123.

49. Castaneda, C.; Gordon, P.L.; Parker, R.C.; Uhlin, K.L.; Roubenoff, R.; Levey, A.S. Resistance Training to Reduce the Malnutrition-Inflammation Complex Syndrome of Chronic Kidney Disease. *Am. J. Kidney Dis*. **2004**, *43*, 607–616.

50. Oliveros, M.S.; Avendaño, M.; Bunout Barnett, D.; Hirsch Birn, S.; Maza Cave, M.P.D.L.; Pedreros, C.; Müller, H. A pilot study on physical training of patients in Hemodialysis Estudio piloto sobre entrenamiento físico durante hemodiálisis. **2011**.
